# Mechanisms of Atrial Fibrillation in Heart Failure: Uncovering Therapeutic Targets in the Atrial Substrate

**DOI:** 10.1007/s11897-026-00744-1

**Published:** 2026-02-19

**Authors:** Danish Sultan, Bianca J. J. M. Brundel

**Affiliations:** Department of Physiology, Amsterdam UMC, Vrije Universiteit, Amsterdam Cardiovascular Sciences, Cardiomyopathy and Arrhythmia, De Boelelaan 1117, Amsterdam, 1081 HV The Netherlands

**Keywords:** Atrial fibrillation, Heart failure, Proteostasis, Inflammasome, CaMKII, TRPC6, Translational therapy

## Abstract

**Purpose of Review:**

Atrial fibrillation (AF) and heart failure (HF) frequently coexist, yet therapeutic progress is hindered by limited understanding of the atrial substrate that sustains AF in HF. This review summarizes key mechanisms through which HF promotes a pro-arrhythmic atrial environment.

**Recent Findings:**

Hemodynamic stress, neurohormonal activation, fibrosis, proteostasis derailment, mitochondrial dysfunction, calcium-handling defects, DNA damage, and inflammation collectively drive atrial structural and electrical remodeling. Although guideline-directed HF therapies and catheter ablation improve outcomes, AF recurrence remains high, underscoring the need to target upstream molecular pathways. Emerging studies identify several druggable mechanisms, including heat shock protein induction, HDAC6-dependent cytoskeletal remodeling, NAD⁺/mitochondrial preservation, PARP1/DNA damage signaling, CaMKII and stress-kinase regulation, NLRP3 inflammasome inhibition, and modulation of mechanosensitive channels such as TRPC6 and Piezo1.

**Summary:**

Targeting the atrial substrate represents a promising strategy to reduce AF burden in HF. Biomarker-guided mechanistic trials and rational combination therapies will be essential to translate these insights into effective treatments.

## Introduction

Atrial fibrillation (AF) is the most prevalent clinical arrhythmia in individuals with heart failure (HF), while HF remains the leading cause of death among patients diagnosed with clinical AF [1]. Approximately 30–40% of patients with HF develop AF during the course of their disease, while up to 50% of those with AF eventually exhibit signs of HF [2]. This bidirectional relationship increases the risk of mortality, stroke, and hospitalization, with a nearly twofold increase in all-cause mortality compared with patients who have either condition alone [1].

Mechanistically, HF triggers AF by creating a pro-arrhythmic atrial substrate through elevated left atrial pressure, neurohumoral activation, oxidative stress, and atrial stretch that collectively drive both electrical and structural remodelling in the atrial myocardium [3, 4]. This progressive atrial remodeling contributes to the development of an atrial cardiomyopathy, a state of electrical, contractile, and structural dysfunction that underlies AF susceptibility and maintenance [5]. Conversely, AF worsens hemodynamic efficiency through loss of atrial contraction, irregular ventricular filling, and tachycardia induced cardiomyopathy [5, 6]. Despite major advances in rhythm control and catheter ablation, recurrence rates of AF remain high in the HF population. The CASTLE-AF trial showed that while catheter ablation improves survival and reduces HF hospitalizations, AF recurrence occurs in up to one-third of patients, consistent with a recent meta-analysis showing atrial tachyarrhythmia recurrence, with residual recurrence rates of approximately 25–30% [7, 8]. This highlights that current therapies fail to address the underlying atrial substrate remodelling, the key driver of AF persistence in the context of HF.

Accordingly, understanding the mechanistic interplay between structural, electrical, molecular, and inflammatory remodelling, within the broader framework of atrial cardiomyopathy, in HF-associated AF is critical to designing substrate directed interventions. This review integrates epidemiological data and mechanistic insights to highlight emerging therapeutic targets capable of breaking the vicious cycle between AF and HF.

## Prevalence and Epidemiology

AF and HF are interrelated cardiovascular epidemics that share common risk factors, including aging, hypertension, obesity, diabetes, and valvular disease, which contribute to myocardial and atrial remodeling. Data from the Framingham Heart Study and Miyasaka et al. demonstrated that the lifetime risk of developing AF in patients with established HF exceeds 40%, while the presence of AF increases the subsequent risk of developing HF by approximately five fold [9, 10].

More recent registry data extend these findings across continents [11]. In the European Society of Cardiology Heart Failure Long-Term Registry (ESC-HF-LT, *n* ≈ 12,000), the prevalence of AF was 37% in the overall cohort, and appeared higher in patients with preserved ejection fraction (HFpEF = 45%) than those with reduced ejection fraction (HFrEF = 32%) [12]. In the ASIAN-HF registry (*n* ≈ 5,000 Asian patients), AF prevalence was 26% in HFrEF and 40% of HFpEF patients underscoring similar ejection-fraction phenotypes and by geographic and ethnic groups [13]. A recent analysis from the Get With The Guidelines (GWTG)-AFIB and GWTG-HF registries linked hospitalized patients enrolled between 2013 and 2019, identifying 1,642 hospitalizations among 1,426 unique individuals with both HF and AF. Patients with HFpEF or HF with mid-range EF (HFmrEF) were typically older (median 80 vs. 72 years), more often female (61% vs. 33%), and predominantly non-Black (95% vs. 84%) compared to those with HFrEF [14].

Similarly, the clinical setting strongly influences observed AF prevalence. Among patients with acute decompensated HF (ADHF), the GWTG-HF registry reported AF in 31% of hospital admissions which was associated with increased in-hospital mortality and prolonged length of stay [15]. Conversely, ambulatory HF cohorts, such as PARADIGM-HF and TOPCAT, reported AF in 21–36% of stable outpatients, where it remained an independent predictor of cardiovascular death and HF hospitalization [16, 17]. In the recent DELIVER trial, which enrolled ambulatory patients with HF with preserved or mildly reduced left ventricular ejection fraction (LVEF > 40% *n* = 6,263), AF was present at baseline in 56.7% of participants (18.0% paroxysmal AF, 38.7% persistent/permanent AF) [18]. Patients with AF experienced higher rates of the primary endpoint (cardiovascular death or worsening HF), driven largely by HF hospitalisations (4.5 versus 7.5 and 6.4 hospitalisations per 100 person for no AF, paroxysmal AF and persistent/permanent AF, respectively) [18].

Across all subtypes, AF confers a substantial incremental risk of stroke, sudden cardiac death, and hospitalization. A comprehensive meta-analysis by Liu et al. (2021), which pooled data from 14 studies including nearly two million HFpEF patients (*n* = 1,948,923), demonstrated that AF was associated with an 11% higher risk of all-cause mortality [19]. Notably, HFpEF patients with AF tend to be older, more hypertensive, and more often female, reflecting a distinct clinical and biological phenotype that may require tailored management strategies [2, 20, 21].

## Pathophysiology and Atrial Remodelling

**Fig. 1:**
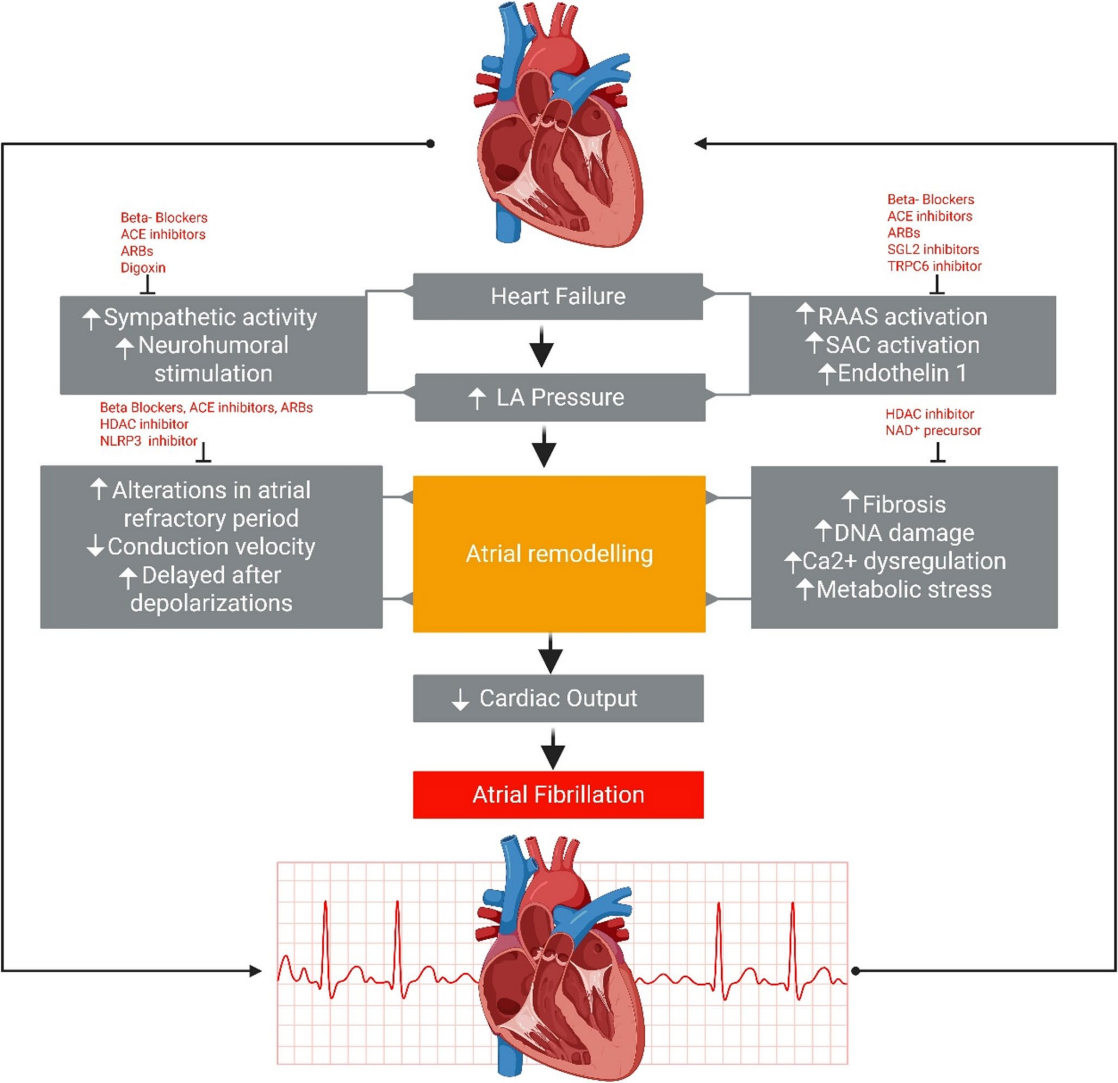
Pathophysiology of atrial fibrillation in heart failure with current and novel drug targets. Heart failure leads to elevated left atrial pressure, increased sympathetic activity, and activation of neurohumoral pathways including the renin–angiotensin–aldosterone system (RAAS) and stretch-activated channels (SACs). These changes promote atrial remodeling, characterized by extracellular matrix remodeling, proteostasis derailment, DNA damage, metabolic stress, and Ca²⁺ dysregulation, which together lead to sustained functional changes such as alterations in atrial refractory periods, reduced conduction velocity, and increased delayed afterdepolarizations. Collectively, these alterations reduce cardiac output and increase susceptibility to AF. RAAS: renin–angiotensin–aldosterone system, SACs : stretch-activated channels, ACE: angiotensin-converting enzyme, ARBs: Angiotensin II Receptor blockers, SGLT2: sodium/glucose cotransporter 2, TRPC6: Transient receptor potential canonical 6, NLRP3: NLR family pyrin domain containing 3. Created with BioRender.com (BioRender, EY290QSW2X)

The coexistence of AF and HF reflects a shared pathophysiological substrate characterized by atrial cardiomyopathy and mechanistically, by mechanical overload, neurohumoral activation, proteostasis derailment, metabolic stress, DNA damage [22] and inflammation. These processes converge to remodel atrial structure and electrophysiology, creating a self-perpetuating cycle in which AF promotes HF progression and vice versa. A key component of this interaction is adverse atrial remodeling, encompassing dilation, fibrosis, myocyte injury, and electrical instability, that underlies AF initiation and maintenance in the failing heart (Fig. 1).

### Hemodynamic Stress and Neurohormonal Activation

AF in HF is strongly influenced by hemodynamic stress and neurohormonal activation, which together create a pro-arrhythmic atrial substrate. Elevated left ventricular filling pressures and chronic volume overload lead to atrial dilation and increased wall stress. These mechanical forces are sensed by Stretch-activated ion channels (SACs) [23, 24], including piezo1 [25, 26] and TRPC6 [27, 28], which elevate intracellular calcium in atrial cardiomyocytes and fibroblasts. The resulting mechano-electrical feedback promotes triggered activity and conduction heterogeneity [29]. In parallel, stretch-induced signaling activates fibroblasts, extracellular matrix deposition, and atrial fibrosis, which further enhances AF susceptibility [23, 27]. Hemodynamic stress in HF also stimulates endothelin-1 (ET-1) synthesis in cardiomyocytes and endothelial cells, and ET-1 exacerbates these stretch-driven processes by enhancing calcium influx, promoting fibroblast activation, and potentiating atrial fibrosis [30–33].

Neurohormonal systems act synergistically with mechanical stress to amplify these maladaptive processes. Activation of the renin-angiotensin-aldosterone system (RAAS) in HF leads to increased angiotensin II and aldosterone levels, which stimulate atrial fibroblast proliferation and collagen synthesis [34, 35]. Angiotensin II also enhances oxidative stress and calcium-handling abnormalities in atrial cardiomyocytes, augmenting the arrhythmogenic potential of the atrium [35]. Similarly, sympathetic nervous system overactivity elevates catecholamine levels, promoting calcium overload, early afterdepolarizations, and abnormal automaticity [36]. Endothelin-1, whose production is also upregulated by RAAS and sympathetic activation, further destabilizes atrial electrophysiology by increasing intracellular calcium, enhancing automaticity, and promoting afterdepolarizations. ET-1 additionally drives profibrotic gene expression, thereby reinforcing both structural and electrical remodeling in HF and in AF [30–33]. Both RAAS and sympathetic activation contribute not only to atrial structural remodeling but also to electrical instability, creating a substrate that supports AF initiation and maintenance [36].

### Fibrosis and Extracellular Matrix Remodeling

Fibrosis is a hallmark of atrial remodeling in HF and is central to AF pathogenesis in these patients [37, 38]. Mechanical stretch, neurohormonal activation, and local inflammatory signaling stimulate fibroblast proliferation and differentiation into myofibroblasts, which produce collagen types I and III and other extra cellular matrix (ECM) proteins [39]. Excess ECM deposition disrupts electrical coupling between cardiomyocytes, increasing conduction heterogeneity and facilitating reentry [40]. Although MRI-based structural imaging of atrial tissue (e.g., late gadolinium enhancement) is frequently interpreted as reflecting atrial fibrosis [41], these signals do not exclusively represent collagen deposition and may also arise from other forms of atrial remodeling, including inflammation or extracellular volume expansion. Interestingly, a study that correlated the extent of atrial fibrosis with electrical conduction abnormalities in AF patients with normal left ventricular function did not find any association between them [42]. Whether fibrosis levels correlate with electrical conduction abnormalities in patients with AF and HF is unknown [43]. However, various molecular changes have been found in AF with HF patients. These changes include oxidative stress, TGF-β signaling, and NLRP3 inflammasome activation, establishing a vicious cycle of structural and electrical dysfunction [36].

###  Proteostasis Derailment

In AF and HF, the proteostasis network becomes disrupted due to sustained mechanical, oxidative, and metabolic stress. Chronic activation of stress pathways, including the heat shock stress response and elevated reactive oxygen species lead to protein misfolding, aggregation, and impaired chaperone function [44]. Key heat-shock proteins (HSPs), especially HSP70, HSP27, and αB-crystallin, are downregulated or functionally exhausted, compromising cytoskeletal stability and ion channel trafficking [45, 46]. Simultaneously, dysfunction of the ubiquitin–proteasome system (UPS) and autophagy allows damaged proteins and organelles to accumulate [47].

This proteostasis derailment contributes to electrical and structural remodeling by destabilizing microtubules, disrupting calcium handling, and impairing mitochondrial and contractile function. In AF, the loss of HSP-mediated protection promotes atrial cardiomyocyte injury and perpetuates arrhythmogenic remodeling [48]. In HF and cardiomyopathy, similar mechanisms underlie progressive myocyte dysfunction and maladaptive remodeling [46, 48]. Chronic oxidative and endoplasmic reticulum stress in HF and in AF overwhelm the heat shock response (HSR), leading to misfolded protein accumulation in atrial cardiomyocytes and fibroblasts [47]. Reduced chaperone activity (e.g., HSP27, HSP70) destabilizes the cytoskeleton, impairs calcium handling, and promotes autophagy [44, 48]. Simultaneously, autophagic protein degradation, a critical mechanism for clearing damaged organelles and misfolded proteins, is often impaired in both HF and AF [49, 50]. Key markers of autophagy, including light chain 3 (LC3)-B and its activated form LC3B-II, are altered in both HF and AF [47, 50], reflecting impaired autophagosome formation and reduced autophagic flux. Defective autophagy promotes accumulation of dysfunctional mitochondria and enhances oxidative stress, which in turn activates calcium/calmodulin-dependent protein kinase II delta (CAMK2D) [51], a key mediator of calcium-handling abnormalities and arrhythmogenic activity [52]. The combined effect of impaired autophagy, altered LC3B signaling, and CAMK2D activation contributes to fibroblast activation, ECM remodeling, calcium dysregulation, and electrical heterogeneity, thereby reinforcing structural and molecular substrates for AF [51, 53]. Restoration of autophagic flux or modulation of CAMK2D activity has been shown in preclinical studies to reduce atrial fibrosis, improve calcium handling, and lower AF susceptibility [47, 53].

### DNA Damage, Calcium Handling and Inflammatory Pathways

Oxidative stress and chronic inflammation in HF and AF induce DNA strand breaks [54–57], triggering activation of poly(ADP-ribose) polymerase-1 (PARP-1), which mediates repair but can deplete NAD⁺ when overactivated [55–58]. PARP-1 overactivation exacerbates energetic stress and mitochondrial dysfunction, promoting apoptosis, cellular senescence, and fibroblast proliferation [57]. This DNA damage-induced PARP1 activation disrupts calcium handling and enhances oxidative signaling, promoting structural and electrical remodeling [57, 59, 60]. Pharmacological or genetic inhibition of PARP1 protects against these abnormalities, reducing arrhythmogenic remodeling and overall AF burden [22, 59].

Additional to DNA damage, a recent study demonstrated that mitochondrial Ca²⁺ accumulation during increased workload is markedly blunted in atrial cardiomyocytes from AF patients. This defect was accompanied by impaired NAD[P]H/FAD regeneration, excessive mitochondrial ROS, and disorganization of nanoscale SR–mitochondrial contacts associated with microtubule destabilization [61]. In human iPSC-derived cardiomyocytes, pharmacologic microtubule disruption displaced mitochondria and increased pro-arrhythmic Ca²⁺ sparks. These abnormalities were rescued by mitochondrial ROS scavenging or restoration of mitochondrial Ca²⁺ uptake (ezetimibe), directly linking cytoskeletal integrity, SR–mitochondrial coupling, mitochondrial energetics, and arrhythmogenesis [61]. Complementary findings identified down-regulation and mislocalization of mitofusin-2 (MFN2) as an additional mechanism disrupting SR–mitochondrial tethering in AF and HF [62–64]. Loss of MFN2 impaired mitochondrial Ca²⁺ uptake, reduced ATP generation, and elevated ROS, culminating in calcium-handling defects and contractile dysfunction. Genetic or pharmacological restoration of MFN2 expression re-established SR–mitochondrial coupling, normalized calcium transients, and mitigated AF inducibility in experimental models, underscoring MFN2 as a potential therapeutic target for stabilizing mitochondrial dynamics and energy homeostasis [62]. Together, these studies extend earlier observations that AF and HF share energy-metabolic dysregulation driven by mitochondrial dysfunction, manifested as ATP depletion, oxidative phosphorylation impairment, and mtDNA damage [65], with the left atrium displaying greater ATP loss and chamber-specific vulnerability [65]. In HF, dysregulated calcium handling, RyR2 oxidation [66], CaMKIIδ overactivation [53, 54], and mitochondrial calcium overload promote delayed afterdepolarizations, conduction heterogeneity, and ectopic triggers, creating a substrate conducive to AF [67, 68]. Molecular regulators, including microRNAs [69–72] and NF-κB signaling, further modulate calcium-handling proteins and fibroblast activity, linking molecular perturbations to electrophysiologic instability [73, 74]. Chronic inflammation in HF drives atrial remodeling through NF-κB activation, NLRP3-inflammasome induction, and pro-fibrotic cytokine release; these inflammatory signals synergize with mechanical stress, neurohormonal activation, and oxidative stress to reinforce fibroblast activation, ECM deposition, and electrical heterogeneity [73, 75–78].

## Current Therapeutic Approaches of AF for Patients with HF

Effective management of AF in patients with HF requires addressing both arrhythmia and ventricular dysfunction, as the interplay between the two conditions perpetuates adverse remodeling and clinical deterioration. Therapeutic strategies thus follow a dual, integrative approach aimed at controlling AF while simultaneously optimizing guideline-directed HF therapy tailored to the severity and chronicity of each condition [34, 47]. Pharmacological agents targeting neurohormonal activation, fibrosis, inflammation, and ion-channel dysregulation remain central, complemented by rate and rhythm control strategies individualized to patient phenotype.

Guideline-directed medical therapy for HF [79, 80], directs the use of angiotensin converting enzyme (ACE) inhibitors, angiotensin-receptor blockers (ARBs), angiotensin receptor neprilysin inhibitors (ARNIs), β-blockers, mineralocorticoid receptor antagonists (MRAs), diuretics, and sodium glucose cotransporter-2 (SGLT2) inhibitors for improved survival and reduced incidence of AF [47]. A meta-analysis of four randomized trials demonstrated that ACE inhibitors and ARBs lowered the relative risk of incident AF by 44% in HF populations, with the greatest benefit observed in those with severely impaired left ventricular function (up to 78% reduction) [81]. These agents attenuate atrial stretch, pressure overload, and RAAS activation, thereby limiting fibrosis and electrical remodeling, a key substrates for persistent AF. Once ventricular rate control is achieved, clinicians must determine whether restoration of sinus rhythm confers additional benefit. Direct current cardioversion and catheter ablation provide principal method for acute rhythm restoration, but AF recurrence remains common (25–30%), and rhythm control strategies has not consistently improved survival compared with rate control alone [7, 8, 80].

Pharmacological rhythm control faces similar limitations, in the AF-CHF trial, amiodarone-based rhythm control offered no advantage over β-blocker–based rate control (with or without digoxin) in reducing cardiovascular mortality or major clinical events [82]. Similarly, AF recurred in 58% of patients in the rhythm-control group and 60–70% in the rate-control group, yet outcomes were comparable, suggesting maintaining sinus rhythm pharmacologically may not improve prognosis, though it can relieve symptoms and improve exercise tolerance [83, 84].

However, the EAST-AFNET 4 trial provided new evidence supporting early rhythm control. In 2,789 patients with newly diagnosed AF (< 12 months), early rhythm control reduced the composite primary endpoint by 22% over a 5.1-year follow-up, driven largely by increased use of catheter ablation. Importantly, the mortality benefit was confirmed in the HF subgroup, suggesting that early rhythm management may offer advantages not observed in earlier pharmacological-only trials [85].

Among available antiarrhythmic agents, only two, amiodarone and dofetilide, are recommended for rhythm control in patients with HFrEF [80]. Class IC agents (flecainide, encainide) and dronedarone are contra-indicated due to increased mortality and/or worsening of HF in decompensated or long-standing AF [80, 86]. In the CHF-STAT trial, amiodarone facilitated conversion to sinus rhythm and was associated with lower mortality [87], though causality remains uncertain; maintaining sinus rhythm might identify a lower risk population rather than directly improving survival. Dofetilide was superior to placebo in maintaining sinus rhythm in HF patients [88, 89], but, similar to amiodarone, offered no mortality advantage and carries a risk of torsades de pointes and other proarrhythmias. These limitations underscore the need for next generation atrial selective ion-channel blockers (e.g., I_Kur or I_KACh inhibitors), or new drugs directed at the molecular mechanisms that drive AF in HF. which may restore atrial electrical stability while minimizing ventricular toxicity.

Rate control remains fundamental to symptom management and ventricular protection. β-blockers are first-line agents for HFrEF and AF, though their mortality benefit is primarily documented in sinus rhythm rather than ongoing AF [90]. Digoxin, frequently combined with β-blockers, assists in rate control, but has been linked to higher mortality rate due to frailty of the HF patients in the cohorts [91].

## Novel Drug Targets

Despite pharmacologic limitations, restoration of sinus rhythm can reestablish atrioventricular synchrony, improve hemodynamic performance, and reverstore tachycardia-induced cardiomyopathy in appropriately selected HFrEF patients [80, 83]. Nonetheless, the absence of consistent survival benefit from drug-based rhythm control highlights an urgent need for mechanism-based therapies, such as drug directed at proteostasis (HSP inducers), NLRP3 inflammasome, or stretch activated ion channels such as TRPC6, to complement conventional approaches. Clinical trials and pre-clinical studies highlight several potential drug targets, which show beneficial effects in AF and HF studies (Table 1; Fig. 1).

**Table 1:** Novel mechanism-based drug targets for atrial fibrillation in heart failure

Pathway	Compound	Mechanism	(Pre-)clinical Stage	Refs or clinical trial identifier
Proteostasis & Heat shock response	Geranylgeranylacetone (GGA)	Induces HSP27/HSP70, reduces ROS, preserves sarcomere integrity	HF: II AF: II	**HF**: NCT05672134 **AF**: [92, 93]
	L-glutamine	Increases HSPs, restores metabolic balance, reduces oxidative/ER stress	HF: II, AF: Preclinical	**HF**: NCT01534663 **AF**: [94]
ER stress modulation	dapagliflozin	SGLT2 inhibitor, Chemical chaperone; alleviates ER stress, improves proteostasis	HF: FDA approved AF: III FDA approved for other uses [95]	**HF**: NCT03619213 NCT03036124 **AF**: NCT01730534
Cytoskeletal remodeling	Tubastatin A (ACY-1215)	Selective HDAC6 inhibitor; restores microtubule acetylation & Ca²⁺ handling	Preclinical in both HF and AF	**HF**: [96] **AF**: [97]
	CKD-504		Preclinical: Huntington’s disease	[98]
	Citarinostat (ACY-241)		Phase: Ib for other conditions	[99, 100]
	TYA-018		HF: I	**HF**: [101, 102]
Mitochondrial and NAD⁺ homeostasis / DNA damage	Nicotinamide riboside	NAD⁺ precursor; restores mitochondrial metabolism, reduces oxidative stress	HF: II AF: Ib	**HF**: NCT02689882 NCT03423342 NCT03727646 **AF**: MEC-2021–0734 [57, 97]
Ca²⁺ handling pathway / kinase regulation	SP600125	JNK inhibitor; prevents CaMKII activation, normalizes SR Ca²⁺ handling	Preclinical in both HF and AF	**HF**: [103] **AF**: [104–106]
Inflammasome and inflammatory cascade	MCC950	Selective NLRP3 inflammasome inhibitor; reduces IL-1β, fibrosis	Preclinical in both HF and AF	**HF**: [78, 107, 108] **AF**: [109, 110]
Stretch-induced remodeling	TRPC6 inhibitor (BI-749327)	Inhibits stretch-activated TRPC6 Ca²⁺ influx, suppresses profibrotic signaling	Preclinical in both HF and AF	**HF**: [111, 112] **AF**: [113]

AF: atrial fibrillation, HF: heart failure, ER: endoplasmic reticulum, HSP: heat shock protein, SGLT2 inhibitor: sodium/glucose cotransporter 2 inhibitor, JNK inhibitor: c-Jun N-terminal kinase inhibitor, TRPC6: Transient receptor potential canonical 6, NLRP3 inhibitor: NLR family pyrin domain containing 3 inhibitor

Among these, agents targeting proteostasis derailment and the heat shock response have shown substantial promise. The HSP-inducer geranylgeranylacetone (GGA) preserved calcium handling L-type Ca²⁺ current, contractility and action potential durations tachypaced and (acute) ischemic canine and HL-1 atrial cardiomyocyte models for AF [93] and in the canine model AF, oral GGA treatment increased atrial HSP expression, prevented shortening of refractoriness, and reduced AF inducibility [93]. Follow-up work with GGA-59, a potent GGA derivative, confirmed that post-treatment restored microtubule stability and contractile protein expression by repressing HDAC6 activity and enhancing HSPB1 and HSPA1 levels in cultured atrial cardiomyocytes and in in human right and left atrial appendages [114, 115]. Similarly, L-glutamine, a naturally occurring amino acid acts as a metabolic substrate that fuels the tricarboxylic acid cycle, enhances HSP expression [116], and suppresses oxidative and ER stress [117]. In phase II HF trial (NCT01534663), glutamine supplementation improved redox balance and mitochondrial function, with preclinical evidence supporting its antiarrhythmic potential in AF [94].

Beyond proteostasis, targeting ER stress has gained traction as a therapeutic avenue. Dapagliflozin, a sodium–glucose cotransporter 2 (SGLT2) inhibitor approved for HF and AF management (NCT03619213, NCT01730534), not only lowers blood glucose but also acts as a chemical chaperone, alleviating ER stress, restoring proteostasis, and stabilizing cardiomyocyte protein folding [118, 119]. These pleiotropic effects likely contribute to its ability to reduce AF incidence in HF patients, linking improved cellular protein handling to rhythm stabilization.

Epigenetic and cytoskeletal remodeling also play pivotal roles in atrial cardiomyopathy. The selective HDAC6 inhibitor tubastatin A (Table 1) restores microtubule acetylation and calcium handling while protecting against contractile dysfunction in experimental AF models [97, 101]. By stabilizing the cytoskeleton, HDAC6 inhibition offers a structural strategy to reverse atrial remodeling distinct from conventional antiarrhythmic agents [97].

At the mitochondrial level, preservation of NAD⁺ homeostasis represents another promising target. Nicotinamide riboside, a vitamin B3 derivative, replenishes cellular NAD⁺ pools, enhances mitochondrial respiration, and reduces oxidative injury and DNA damage [57]. In phase II HF trials (NCT02689882, NCT03423342, NCT03727646), nicotinamide riboside safely increased systemic NAD⁺ levels, while preclinical AF models showed attenuation of electrical remodeling and oxidative damage [57].

In terms of electrical remodeling, inhibition of stress-activated kinases offers another mechanistic route. The JNK inhibitor SP600125 prevents CaMKII hyperactivation and normalizes SR Ca²⁺ handling, thereby reducing triggered activity and AF inducibility in preclinical models [85–88]. Complementing this, suppression of inflammation has emerged as a key antiarrhythmic mechanism. The NLRP3 inflammasome inhibitor MCC950 demonstrated robust antifibrotic and anti-inflammatory effects in animal models of AF and HF, providing proof of concept for inflammasome blockade as a disease-modifying strategy [89–93].

Finally, atrial stretch and mechanotransduction, which are central to HF-induced atrial remodeling, are being targeted through mechanosensitive ion channel modulation. Inhibiting TRPC6 with compounds such as BI-749,327 suppresses stretch induced Ca²⁺ influx, reduces profibrotic signaling, and limits atrial fibroblast activation in preclinical models [94–96].

Collectively, these emerging compounds represent a new generation of mechanism-based interventions poised to transform the treatment approaches of AF in HF.

## Conclusion

AF and HF exist in a vicious cycle of structural, electrical, and metabolic remodeling that drives progressive atrial cardiomyopathy, dysfunction, hemodynamic compromise, and adverse outcomes. Despite advances in guideline-directed therapy and rhythm control, current treatments remain largely symptomatic and fail to modify the atrial substrate underlying arrhythmia inducibility in the setting of HF.

Recent mechanistic insights reveal that atrial remodeling in HF extends beyond traditional neurohormonal and hemodynamic stress, encompassing oxidative injury, proteostasis derailment, mitochondrial dysfunction, and inflammatory signaling. These interconnected pathways not only sustain the arrhythmogenic substrate but also identify novel therapeutic drug targets. Pharmaceutical compounds that restore proteostasis (e.g., HSP inducers), normalize calcium handling (e.g., CaMKII and JNK inhibitors), attenuate inflammation (e.g., NLRP3 blockade), and counteract stretch-induced signaling (e.g., TRPC6 inhibition) represent a new paradigm of substrate directed therapy.

Future research should focus on translating these mechanistic discoveries into precision interventions or combination therapies capable of reversing atrial remodeling and improving clinical outcomes in patients with AF and HF. Integrating molecular biomarkers, high resolution imaging, and electrophysiologic mapping into clinical trials will be essential to bridge the gap between experimental promise and therapeutic reality.

## Key References

- Pronto JRD et al., Impaired atrial mitochondrial calcium handling in human AF. Circ Res 2025. https://doi.org/10.1161/CIRCRESAHA.124.325658.
  - ○ This high-impact human study identifies disrupted SR–mitochondrial coupling and mitochondrial Ca²⁺ dysregulation as primary energetic defects in AF, bridging cytoskeletal instability, mitochondrial stress, and arrhythmogenesis. It provides the strongest evidence that mitochondrial dysfunction is a central, not secondary, feature of AF in HF.
- Zhang D. et al., DNA damage–induced PARP1 activation drives NAD⁺ depletion and atrial remodeling. Nat Commun 2019. https://doi.org/10.1038/s41467-019-09014-2.
  - ○ This seminal study demonstrates that DNA damage–PARP1 signaling is a causative and druggable driver of AF-related remodeling. It establishes the mechanistic link between oxidative stress, NAD⁺ depletion, mitochondrial dysfunction, and arrhythmogenesi.
- Cheng X. et al. NLRP3 inflammasome inhibition by MCC950 attenuates cardiac and pulmonary artery remodeling in HFpEF. Life Sci. 2023. https://doi.org/10.1016/j.lfs.2023.122185.
  - ○ Shows that NLRP3 activation drives HFpEF-related inflammation and fibrosis; MCC950 reverses remodeling. Establishes NLRP3 as a mechanistic and therapeutic target relevant to AF-HF overlap.
- Brundel BJJM et al. (2022) — Nature Reviews Disease Primers overview Atrial fibrillation. Nat Rev Dis Primers. 2022. https://doi.org/10.1038/s41572-022-00347-9.
  - *○*Authoritative, high-impact synthesis describing AF as a multi-system proteostasis, metabolic, inflammatory, and structural disorder. This review provides the conceptual backbone supporting the mechanistic focus of your manuscript.

## Data Availability

No datasets were generated or analysed during the current study.
